# The Labile Iron Pool Reacts Rapidly and Catalytically with Peroxynitrite

**DOI:** 10.3390/biom11091331

**Published:** 2021-09-09

**Authors:** André Luís Condeles, José Carlos Toledo Junior

**Affiliations:** Departamento de Química, Faculdade de Filosofia, Ciências e Letras de Ribeirão Preto, Universidade de São Paulo, Ribeirão Preto 14040-901, Brazil; acondeles@usp.br

**Keywords:** labile iron pool, nitric oxide, superoxide, peroxynitrite, oxidative stress, nitrogen dioxide

## Abstract

While investigating peroxynitrite-dependent oxidation in murine RAW 264.7 macrophage cells, we observed that removal of the Labile Iron Pool (LIP) by chelation increases the intracellular oxidation of the fluorescent indicator H_2_DCF, so we concluded that the LIP reacts with peroxynitrite and decreases the yield of peroxynitrite-derived oxidants. This was a paradigm-shifting finding in LIP biochemistry and raised many questions. In this follow-up study, we address fundamental properties of the interaction between the LIP and peroxynitrite by using the same cellular model and fluorescence methodology. We have identified that the reaction between the LIP and peroxynitrite has catalytic characteristics, and we have estimated that the rate constant of the reaction is in the range of 10^6^ to 10^7^ M^−1^s^−1^. Together, these observations suggest that the LIP represents a constitutive peroxynitrite reductase system in RAW 264.7 cells.

## 1. Introduction

Iron can coordinate with different types and numbers of ligands to form compounds with different geometries, while reversibly adopting a few stable oxidation and spin states. Such versatility provides iron compounds with different structural and chemical properties, which allow them to engage in fundamentally distinct chemical reactions. In particular, iron-mediated redox reactions are central in numerous biological processes, from biosynthesis to catabolism and energy transduction [1]. The iron pool associated with these physiological functions is firmly and directly bound to proteins or incorporated into protein prosthetic cofactor groups such as heme and iron sulfur-clusters. In contrast, the so-called labile iron pool (LIP) is poorly characterized, but there is growing evidence that the LIP is also protein-bound.

The LIP is defined as the fraction of cellular iron that can be complexed with high-affinity metal chelators [1,2]. Depending on the cell type, the iron content of this pool accounts for 0.1 to 3% of the total cellular iron. Perturbations in the levels of the LIP trigger cellular iron homeostasis mechanisms involving complementary actions that work toward restoring the status of the LIP [3]. For example, a manipulated rise in the LIP in K562 cells was found to be transient and to dissipate within 30 min [4]. These observations indicated that the LIP is under tight cellular homeostatic control and is an essential cellular component [2,3,5]. Currently, the LIP is thought to be primarily present in the ferrous state [4] and serve as a dynamic cellular iron source readily available for incorporation into fresh apo-iron proteins [3]. On the other hand, the LIP is often associated with an elusive cellular iron source that can potentially damage metabolites, macromolecules, and macro-biological structures through reaction with hydrogen peroxide (H_2_O_2_), which produces oxidants such as the highly reactive hydroxyl radical (OH^•^) [4,6]. This property is being explored as a potential cancer therapy [7,8]. Indeed, the LIP is often referred to as the redox toxic iron source developing from conditions such as iron overload, and it has been associated with hepatic [9,10,11] and cardiac [12,13] conditions and brain disorders [3,14,15,16]. Thus, the same redox activity that makes iron so physiologically useful may also be dangerous. The central importance of iron in biological systems, its intrinsic toxicity and elusive nature have motivated investigations into the chemical properties and reactivity of the LIP. Previously, our group [17] and others [18,19,20] showed that the LIP reacts with nitrogen monoxide (nitric oxide, NO^•^), producing dinitrosyl iron complexes (DNICs) in RAW 264.7 cells, which are considered stable NO^•^ carriers. DNICs are potential nitrosylating agents and may directly transfer nitrosyl equivalents to protein cysteine residues via cross-trans-nitrosation reactions [18,21,22], which may be a selective mechanism of protein S-nitrosylation. More recently, we investigated the possible reaction between the LIP and peroxynitrite (ONOOH/ONOO^−^; pKa = 6.9) in RAW 264.7 cells [23,24]. We selected the macrophage cell line as model for this study for several reasons: (i) macrophages cells are thought to be normally exposed to and to deal with peroxynitrite; (ii) macrophage cells are major players in the systemic iron homeostatic mechanism; (iii) macrophage cells normally carry larger concentrations of LIP. Peroxynitrite is formed by the diffusion-limited recombination reaction of ubiquitous NO^•^ and superoxide anion radicals $\left(O\frac{•}{2}\right)$ (k_1_ = 1.9 × 10^10^ M^−1^s^−1^) [24]. In addition to being a strong oxidant, upon protonation or reaction with CO_2_, peroxynitrite generates highly reactive oxidant species such as OH^•^, nitrogen dioxide $\left({\mathrm{NO}}_{2}^{•}\right)$, and carbonate anion radicals $\left(\mathrm{CO}\frac{•}{3}\right)$) [25,26], which can damage various biological molecules and have been implicated in the onset of several pathological conditions [25,27].

**Scheme 1:**
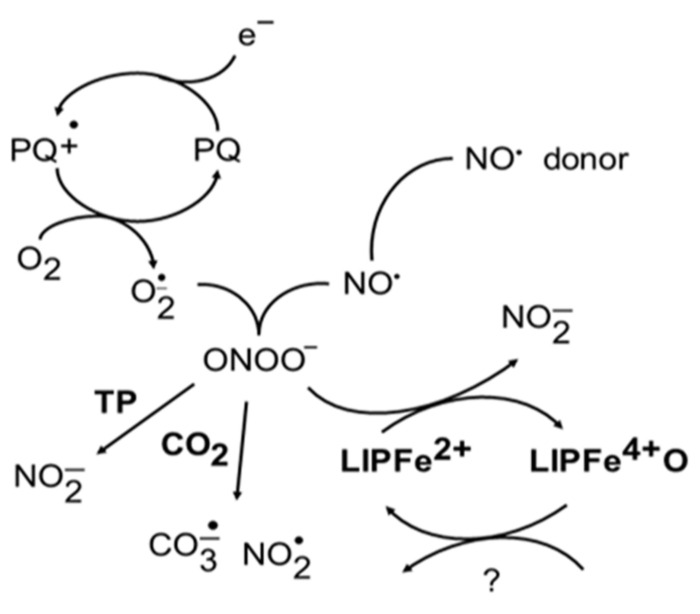
The cellular study model and the hypothetical reaction of the LIP with peroxynitrite. Peroxynitrite is delivered intracellularly by co-production of $O\frac{•}{2}$ through paraquat (PQ) and NO^•^ (NO^•^ donor). Peroxynitrite reacts directly with thiol peroxidases, metalloproteins (TP) and CO_2_, phenomenologically producing 2/3 of ${\mathrm{NO}}_{3}^{-}$ and CO_2_ and 1/3 of the oxidant radicals ${\mathrm{NO}}_{2}^{•}$ and $\mathrm{CO}\frac{•}{3}$. The acid-catalyzed decomposition of peroxynitrite phenomenologically produces 2/3 of ${\mathrm{NO}}_{3}^{-}$ and 1/3 of the oxidant radicals ${\mathrm{NO}}_{2}^{•}$ and OH^•^, but it is kinetically less favorable and has been omitted for simplicity. Hypothesis: The ferrous LIP (LIPFe^2+^) directly reacts with peroxynitrite, presumably producing ${\mathrm{NO}}_{2}^{-}$. This reaction is inhibited by chelation of the LIP, which increases the availability of peroxynitrite and the net yield of peroxynitrite-derived radicals ${\mathrm{NO}}_{2}^{•}$ and $\mathrm{CO}\frac{•}{3}$, enhancing the peroxynitrite-dependent direct and indirect oxidation of cellular targets.

Unlike the reaction between the LIP and H_2_O_2_, we previously demonstrated that the reaction between the LIP and peroxynitrite decreases the peroxynitrite-dependent intracellular oxidation and nitrosylation of fluorescent targets and protein carbonylation [23]. Inspired by the two-electron reduction of peroxynitrite by manganese (II) and iron (II) porphyrin complexes [28,29] as well as ferrous heme proteins [30,31,32], we hypothesized that the ferrous LIP directly reduces peroxynitrite to nitrite $\left({\mathrm{NO}}_{2}^{-}\right)$ (Scheme 1) [23], thereby detoxifying peroxynitrite. However, whether this reaction is rapid and stoichiometric or catalytic is unclear. In the present study, we address these fundamental questions. We will show that our results are consistent with a rapid and catalytic reaction between the LIP and peroxynitrite.

## 2. Materials and Methods

### 2.1. Chemicals

Unless specified otherwise, all the chemicals were purchased from Sigma-Aldrich (St. Louis, Missouri, USA) and were of the highest purity available. The chelator salicylaldehyde isonicotinoyl hydrazine (SIH) was synthesized through a Schiff base condensation reaction between 2-hydroxybenzaldehyde and isonicotinic acid hydrazide, as described previously (ref ponka). The nitric oxide donors and coumarin-7-boronic acid (CBA) were purchased from Cayman Chemical Co (Ann Arbor, MI, USA), and Calcein was acquired from Biotium (Fremont, CA, USA). All the stock solutions including the solutions of NO^•^ donors, Ebselen, hexacyanoferrate (II), ascorbate, and SIH were prepared, maintained, and quantified as described previously [23]. The stock solutions of paraquat were spectrophotometrically calibrated by measuring their absorbance at 600 nm (ε = 2.9 × 10^5^ L mol^−1^ cm^−1^) after reduction with 1–10% of dithionite in 0.1 mol L^−1^ NaOH [33,34].

### 2.2. Cell Culture and Treatment

RAW 264.7 cells (ATCC) were incubated and cultured at 37 °C in Dulbecco’s Modified Eagle’s Medium supplemented with 100 units/mL penicillin, 100 μg/mL streptomycin-penicillin, and 10% fetal bovine serum (FBS). The cells were passaged, seeded onto 75 cm^2^ T-flask culture dishes, and grown overnight, to reach between 85% and 90% confluence. Then, the cells were washed with PBS/DTPA (100 µM) twice, harvested, and centrifuged at 450× *g* and 4 °C for 5 min. Finally, the cells were suspended in 10 mL of complete medium and incubated on ice. The trypan blue exclusion assay was conducted before and after the experiments, and cell viability ranged from 85% to 95%.

### 2.3. Quantification of the LIP

**Figure 1:**
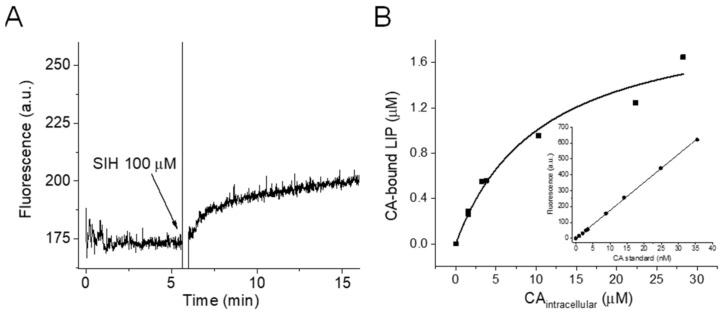
Quantification of the LIP in RAW 264.7 cells. (**A**). Fluorescence trace of Calcein-AM-loaded RAW 264.7 cells upon introduction of SIH. Pre-warmed 3-mL suspensions of 15 × 10^6^ cells previously treated with Calcein-AM (0.25, 0.5, 1.0, 2.0, or 3.0 µM) at 37 °C for 20 min were placed in the fluorimeter. When the baseline was established, SIH (100 µM) was added, as designated in the plot. The conditions and fluorescence acquisition parameter settings were as follows: PBS, 100 µM DTPA, pH 7.4, T = 37 °C, λex = 495 nm, λem = 516 nm, ex and em slit width = 3 nm. (**B**). Intracellular concentration of the CA-bound LIP as a function of the intracellular concentration of free CA. The concentration of the LIP was taken as the limiting concentration of the CA-bound LIP and was determined by fitting the data of Figure 1A to a hyperbolic equation with the aid of Origin 2019 (Academic), OriginLab Corporation, Northampton, MA, USA. The intracellular concentrations of free CA and the CA-bound LIP were determined by unit transformation; the concentrations of the solutions of CA and the CA-bound LIP were determined with the help of a standard analytical curve of fluorescence versus known concentrations of free CA (inset) in control Calcein-AM untreated cells (15 × 10^6^ cells in 3 mL) and in the presence of SIH under the same conditions of the experiments. The CA assays were performed in a conventional spectrofluorometer (Shimadzu RF-5301pc).

Briefly, a suspension of RAW 264.7 cells in PBS/DTPA (45 × 10^6^ cell/mL) was treated with the aceto-methoxy derivatized calcein probe (Calcein-AM) (0.25, 0.5, 1.0, 2.0, and 3.0 μM) under constant stirring at 37 °C for 20 min, washed (two cycles of centrifugation and resuspension in ice-cold CA-free PBS), and kept on ice until use. Calcein-AM is cleaved by nonspecific esterases, forming the fluorescent product Calcein (CA), which can no longer freely cross biological membranes and accumulates intracellularly. At the time of the experiment, a suspension of Calcein-AM-loaded cells (15 × 10^6^ cells in a total volume of 3.0 mL) was transferred to pre-warmed (37 °C) PBS/DTPA and placed in the fluorimeter, and data acquisition was initiated. The initial fluorescence is proportional to the intracellular concentration of free CA. Then, the high-affinity membrane-permeable iron chelator SIH was added with the help of a Hamilton syringe, and the fluorescence signal increased because CA was released (Figure 1A). This differential fluorescence recovery (ΔF) was proportional to the intracellular concentration of the CA-bound LIP. The concentrations of the solutions of free CA and the CA-bound LIP were determined by using a standard analytical curve of fluorescence versus concentration of free CA (Figure 1B inset), which was generated by successive additions of known concentrations of free CA to a suspension of control cells in the presence of SIH. The intracellular concentrations of free CA and the CA-bound LIP were calculated by unit conversion; the following equation was used: ((CA) or (LIP-bound CA-)) × assay total volume/total volume of cells (the diameter of RAW 264.7 cells is 7 μm, as reported elsewhere) [35]. The stock solution of CA was prepared in DMSO, and the concentration was determined by using the absorbance at 492 nm and the molar absorptivity coefficient of ε_492_ = 7.5 × 10^4^ M^−1^ cm^−1^ (provided by Biotium). The total concentration of the LIP in the RAW 264.7 cells was assumed to be the limiting concentration of the CA-bound LIP.

### 2.4. Fluorescence Experiments

A suspension of RAW 264.7 cells in PBS/DTPA was loaded with 30 μM 2′,7′-dichlorodihydrofluorescein diacetate (H_2_DCF-DA; fluorescent indicator) under constant stirring at 37 °C for 30 min. To minimize differences in the intracellular concentrations of the fluorescent indicator during the experiments, the same number of cells was systematically used when the indicator was loaded (60 × 10^6^ cells/mL). Once the indicator permeates biological membranes, the ester bonds present in H_2_DCF-DA are cleaved by non-specific esterases, and the product 2′,7′-dichlorodihydrofluorescein (H_2_DCF) is trapped and begins to accumulate intracellularly. To avoid the presence of extracellular H_2_DCF, the cells were subjected to two cycles of centrifugation and resuspension after the loading procedure. For the assays regarding H_2_DCF, the cells were transferred to 96-well plates (3 × 10^6^ cells per pool in 250 μL), and the experiments were performed in a plate reader (Molecular Device SpectraMax i3x) with the following fluorescence acquisition parameter settings: λexcitation = 498 nm, λemission = 523 nm (H_2_DCF-DA), and excitation and emission slit widths of 9 and 15 nm, respectively. The CBA experiments were performed by using the same procedure, except that CBA (10 µM) was not loaded into the cells but simply included in the PBS/DTPA cellular suspension before the runs. COH and DCF fluorescence was measured at the time intervals designated in the figures. The donors sperNO and deta/NO were the NO^•^ sources in these experiments (these donors have half-lives of 39 min and 20 h at 37 °C and pH 7.4, respectively [36]) as described in the respective figure legends. All the experiments were performed in PBS buffer supplemented with 100 µM DTPA, and the temperature was controlled by a circulating water bath and/or the instrument internal temperature control (heated air flow) maintained at 37 °C.

### 2.5. In the Rate of Fluorescence Increase

The rates at which the intracellular fluorescence of COH and DCF increased were determined by linear regression analyses of the fluorescence data. See the respective figures for more details.

### 2.6. Statistical Analysis

All the measurements are presented as the means ± S.D. of *n* ≥ 3 experiments. The means were compared between groups by using an F test followed by a paired Student’s t-test; the academic version of the software Origin 2019 was employed (OriginLab Corporation, Northampton, MA, USA). *p* values of <0.05 were considered to be statistically significant.

## 3. Results

### 3.1. Quantifying the LIP

First, we evaluated the LIP content of RAW 264.7 cells by using a modified Calcein methodology [2]. Briefly, we pre-loaded a suspension of RAW 264.7 cells in PBS/DTPA (45 × 10^6^ cell/mL) with the aceto-methoxy derivatized calcein probe (Calcein-AM). Calcein-AM is cleaved by nonspecific esterases, forming the fluorescent product Calcein (CA), which can no longer freely cross biological membranes and thus accumulates intracellularly. The intracellular fluorescence of CA is quenched when the LIP binds to its EDTA-like moiety in a 1:1 stoichiometry; the remaining initial fluorescence is proportional to the intracellular concentration of free CA (Figure 1A). When the high-affinity membrane-permeable iron chelator SIH is introduced, the fluorescence signal increases due to LIP and the chelator complexation and CA release (Figure 1A) [2,37]; the differential fluorescence recovery (ΔF) is proportional to the intracellular concentration of the CA-bound LIP. We determined the intracellular concentrations of the CA-bound LIP with increasing intracellular concentrations of free CA (Figure 1A) and fitted the data ((CA-bound LIP) × (free CA)) displayed in Figure 1B to a hyperbolic equation. We assumed that the total concentration of the LIP in RAW 264.7 cells was the limiting concentration of the CA-bound LIP, which was 2.0 ± 0.2 µM.

### 3.2. Checking Formation of Peroxynitrite

Next, we checked the formation of peroxynitrite in cells by co-production of NO^•^ and $O\frac{•}{2}$ according to Scheme 1 [38]. We transferred a suspension of RAW 264.7 cells to 96-well plates and treated it with the NO^•^ donor (*Z*)-1-[*N*-[3-aminopropyl]-*N*-[4-(3-aminopropylammonio)butyl]-amino]diazen-1-ium-1,2-diolate (sperNO) and the redox cycler *N*,*N*′-dimethyl-4,4′-bipyridinium dichloride (paraquat), which catalytically generates intracellular $O\frac{•}{2}$ at the expense of cellular reducing agents, Scheme 1. The combination of paraquat and sperNO is herein referred to as PQ/NO^•^.

**Figure 2:**
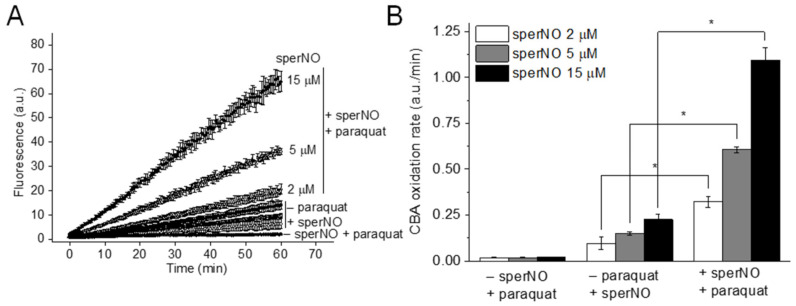
Formation of peroxynitrite by paraquat and NO^•^ donor in cells. Briefly, suspensions of RAW 264.7 cells were transferred to 96-well plates (1.2 × 10^7^ cells/mL). CBA (10 μM), paraquat (10 μM), and sperNO (2, 5 or 15 µM) were individually added to selected wells, in this order. (**A**). COH fluorescence as a function of time. The measurements were initiated immediately after sperNO was introduced, and the fluorescence was registered every minute for one hour. The data represent the mean ± SD (*n* = 4). (**B**). Increase in the rate of COH fluorescence. This parameter was determined by linear regression of the fluorescence data presented in panel A within the first 10 min of each run. The data represent the mean of four independent experiments ± S.D. and are statistically significant at the 95% confidence interval relative to control cells * (*p* < 0.05, Paired Student’s t-test). The assays were performed in PBS, DTPA (100 µM), at pH 7.4 and 37 °C, and the fluorescence acquisition parameters were λex = 332 nm, λem = 456 nm, and ex and em slit width = 9 and 15 nm, respectively.

We followed the formation of peroxynitrite by PQ/NO^•^ in RAW 264.7 cells by fluorescence spectroscopy, by using 10 µM of the boronate compound coumarin-7-boronic acid (CBA). This compound reacts with peroxynitrite at a high rate constant (*k* = 1.1 × 10^6^ M^−1^s^−1^) [39], producing the fluorescent product 7-hydroxy coumarin (COH). As shown in Figure 2, on the basis of the accumulated COH, treatment with PQ/NO^•^ produced peroxynitrite in cells in a sperNO concentration-dependent way at least up to 15 µM sperNO, showing no sign of $O\frac{•}{2}$ exhaustion. This behavior was expected given that NO^•^ and cellular SODs compete for $\left(O\frac{•}{2}\right)$. We used the combination PQ/NO^•^ throughout the study to deliver peroxynitrite to cells.

### 3.3. Monitoring Peroxynitrite-Dependent Oxidation

**Figure 3:**
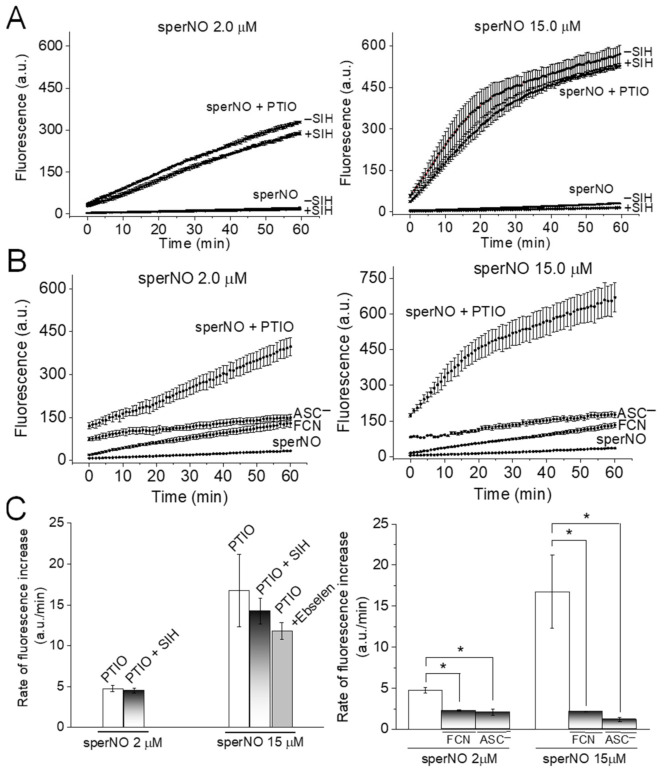
${\mathrm{NO}}_{2}^{•}$-dependent intracellular oxidation of H_2_DCF and the effects of SIH and Ebselen. A suspension of H_2_DCF-loaded RAW 264.7 cells was transferred to 96-well plates (1.2 × 10^7^ cells/mL) and treated with sperNO (2 or 15 μM) in the presence of PTIO (250 μM) plus or minus SIH (100 µM). (**A**)**.** Fluorescence traces of the oxidation of H_2_DCF by ${\mathrm{NO}}_{2}^{•}$ generated by combination of sperNO and PTIO; (**B**)**.** Fluorescence traces of the oxidation of H_2_DCF by ${\mathrm{NO}}_{2}^{•}$ generated by combination of sperNO and PTIO in the presence of the ${\mathrm{NO}}_{2}^{•}$ scavenger potassium hexacyanoferrate (II) [55] or ascorbate (100 μM). The data in panels A and B represent the mean ± S.D of three experiments and are statistically significant at the 95% confidence interval relative to control cells * (*p* < 0.05, Paired Student’s *t*-test); (**C**)**.** Rate of the ${\mathrm{NO}}_{2}^{•}$-dependent intracellular oxidation of H_2_DCF shown in (**A**) and (**B**). The data represent the mean of three independent experiments ±S.D. The assays were performed in PBS and DTPA (100 μM), at pH 7.4, and 37 °C, and the fluorescence acquisition parameters were λex = 498 nm, λem = 523 nm, and ex and em slit width = 9 and 15 nm, respectively.

**Figure 4:**
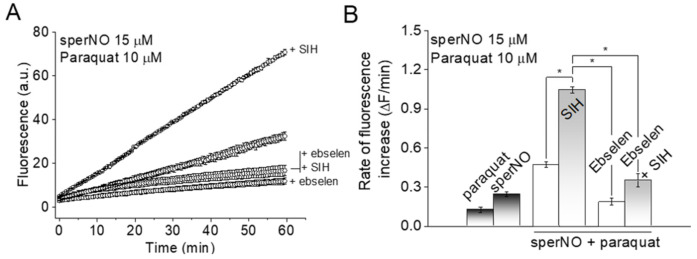
Sequestration of the LIP by chelation increases the peroxynitrite-dependent intracellular oxidation of H_2_DCF. (**A**) Fluorescence traces of the formation of DCF in cells treated with PQ/NO^•^ in the presence and absence of Ebselen and SIH; Briefly, H_2_DCF-loaded RAW 264.7 cells were transferred to 96-well plates (1.2 × 10^7^ cells/mL). Paraquat (10 μM), SIH (100 μM), and Ebselen (50 μM) were individually added to selected wells in this order. The fluorescence measurements were initiated immediately after sperNO (15 μM) was introduced and registered every minute for one hour. Each trace represents the mean ± standard deviation (S.D.) of four experiments. (**B**) The rate at which the fluorescence of DCF increased. This parameter was determined by linear regression of the fluorescence data presented in panel A, by using the data collected during the first 10 min of each run. The data represent the mean of four independent experiments ± S.D. and are statistically significant at the 95% confidence interval relative to control cells * (*p* < 0.05, Paired Student’s *t*-test). The assays were performed in PBS and DTPA (100 μM), at pH 7.4, and 37 °C. The conditions and fluorescence acquisition and parameter settings were the same as those described in Figure 3.

Similarly to our previous study [23], we used 2′,7′-dichlorodihydrofluorescein diacetate (H_2_DCF-DA) to monitor peroxynitrite-dependent oxidation. We visualized that the LIP reacted with peroxynitrite by the incremental peroxynitrite-dependent oxidation of H_2_DCF-DA in the presence of the LIP chelator, which bound to the LIP and inhibited the reaction between the LIP and peroxynitrite. Using H_2_DCF for our purposes has several advantages. First, by using its diacetate form (H_2_DCF-DA), H_2_DCF can be loaded into cells (through action of nonspecific esterases) [23] and accumulates to hundreds of µM intracellularly. This is critical for our goals, given that H_2_DCF has to compete with multiple cellular targets for the peroxynitrite-derived radicals. In our hands, the intracellular concentration of H_2_DCF approached 400 µM in the typical cell loading procedure (Supplementary Material, Figure S1). Second, H_2_DCF does not react directly with the peroxynitrite precursors NO^•^ and $O\frac{•}{2}$ or H_2_O_2_ (the product of $O\frac{•}{2}$ dismutation). Third, H_2_DCF reacts with all peroxynitrite-derived radicals such as OH^•^, ${\mathrm{NO}}_{2}^{•}$, and $\mathrm{CO}\frac{•}{3}$ at high rate constants. If we consider that GSH is the only competitor, we can calculate that 400 µM H_2_DCF reacts with 80% of $\mathrm{CO}\frac{•}{3}$ (assuming 5 mM GSH, *k_GSH_* = 5.3 × 10^6^ M^−1^s^−1^, and *k_H_*_2*DCF*_ = 2.6 × 10^8^ M^−1^s^−1^) [40]. Similar calculations showed that H_2_DCF would react with only 5% of the ${\mathrm{NO}}_{2}^{•}$ (*k_H_*_2*DCF*_ = 1.3 × 10^7^ M^−1^s^−1^) [40], but we confirmed that H_2_DCF efficiently (directly or indirectly) detected ${\mathrm{NO}}_{2}^{•}$ (Please, see Figure 3 below). To guarantee that the oxidation of H_2_DCF depended on peroxynitrite as intermediate, we ran parallel control experiments in the presence of 2-phenyl-1,2-benzoselenazol-3-one (Ebselen), a compound that directly and rapidly reduces peroxynitrite to ${\mathrm{NO}}_{2}^{-}$ (Equation (1)) [41]. Ebselen fully inhibited the intracellular oxidation of H_2_DCF by treatment with PQ/NO^•^ in both the absence and presence of the chelator (Please see Figure 3 and Figure 4 below), indicating that formation of dichloro-fluorescein (DCF) in the presence of PQ/NO^•^ depended on peroxynitrite acting as intermediate. In addition, along with proper controls, H_2_DCF has been used to detect peroxynitrite [42] and to determine fluxes of peroxynitrite in activated macrophages [38].

(1) $$\mathrm{Ebselen}+{\mathrm{ONOO}}^{-}\;\overset{{k\;=\;1\;\times \;10}^{6}{\;M}^{-1}s^{-1}}{\to }\;\mathrm{Selenide}+{\mathrm{NO}}_{2}^{-}$$

The mechanism through which H_2_DCF is oxidized deserves a final comment. Strong radical oxidant species such as those derived from peroxynitrite [40] or high-valent ferryl species derived from reactions of hydrogen peroxide with heme-proteins and heme-peroxidases (known as compound I and compound II) [40,43] oxidize H_2_DCF mono-electronically, yielding the putative radical DCFH^•^. The latter may dismutate, but it more likely reduces O_2_ to $O\frac{•}{2}$, yielding the fluorescent product DCF in the process (supplementary information). Obviously, production of $O\frac{•}{2}$ by this mechanism raises concerns because it could react with NO^•^, to form peroxynitrite, potentially self-stimulating the peroxynitrite-dependent oxidation of H_2_DCF. However, the amount of $O\frac{•}{2}$ (and thus H_2_O_2_) produced by this mechanism is arguably negligible compared to $O\frac{•}{2}$-derived paraquat. According to a rough estimation based on kinetic competition (Supplementary information), $O\frac{•}{2}$-derived H_2_DCF amounts to, at most, less than 6% of the $O\frac{•}{2}$-derived paraquat.

It could be argued that boronate compounds are a more appropriate choice of indicator to investigate the hypothetical reaction between the LIP and peroxynitrite because these compounds directly and rapidly react with peroxynitrite [44,45]. However, such approach poses difficulties. First, only a small fraction of the available peroxynitrite actually reacts with boronate in cells. In a similar cellular model, fluorescein-derived boronate (Fl-B) at a presumed concentration of 50 μM was estimated to react with only 10% of the available peroxynitrite. Moreover, possibly only a small fraction of available peroxynitrite (and even less in the presence of boronates) reacts with the LIP in cells given that the concentration of the LIP is in the low µM range. Rationally, in the presence of the LIP chelator, most peroxynitrite that would otherwise react with the LIP would not react with boronate but with other direct targets of peroxynitrite, such as TP and CO_2_ [46]. Thus, arguably, inhibition of the reaction between the LIP and peroxynitrite by the chelator would cause only a small and possibly experimentally undebatable increase in the peroxynitrite-dependent oxidation of boronate. We did not detect any increase in the oxidation of Fl-B in the presence of a chelator (not shown) in cells challenged with PQ/NO^•^.

### 3.4. H_2_DCF Efficiently Detected Different Levels of ${\mathrm{NO}}_{2}^{•}$ in Cells Regardless of the Presence of the Chelator SIH

To test whether H_2_DCF reacted with peroxynitrite-derived radicals under our experimental conditions, we had to design a strategy to deliver these species specifically to the cellular suspension model. We selected ${\mathrm{NO}}_{2}^{•}$ as the prototype species. The ${\mathrm{NO}}_{2}^{•}$ radical is a freely diffusible species that can rapidly access the cytosolic space [47,48], and it reacts rapidly with H_2_DCF (*k* = 1.3 × 10^7^ M^−1^s^−1^) [40]. To the best of our knowledge, no currently available method can specifically deliver a continuous and sustained flux of the other peroxynitrite-derived species OH^•^ and $\mathrm{CO}\frac{•}{3}$ in the absence of other oxidants in cells. On the other hand, we generated increasing fluxes of ${\mathrm{NO}}_{2}^{•}$ practically devoid of NO^•^ and other oxidants by mixing different concentrations of NO^•^ donor with excess 2-phenyl-4,4,5,5-tetramethylimidazoline-1-oxyl 3-oxide (PTIO), an imidazoline-oxyl N-oxide that oxidizes NO^•^ to ${\mathrm{NO}}_{2}^{•}$ [49,50,51,52,53,54] (Supplementary Material, Figure S2). We performed the experiment as follows. We placed H_2_DCF-loaded RAW 264.7 cells in 96-well plates and challenged them with sperNO in the presence of 250 µM PTIO, in the absence and in the presence of the chelator SIH (Figure 3A). As expected, compared to sperNO alone, PTIO, which fully oxidized NO^•^ to ${\mathrm{NO}}_{2}^{•}$, dramatically increased the rate at which DCF was formed. SIH had no effect on the H_2_DCF oxidation under these conditions. That ${\mathrm{NO}}_{2}^{•}$ was the H_2_DCF oxidant in these experiments was confirmed by performing experiments in the presence of rapid ${\mathrm{NO}}_{2}^{•}$ scavengers, hexacyanoferrate (II) (FCN) [55,56] and ascorbate (ASC^−^) [57]. Neither FCN nor ASC^−^ reacts rapidly or at all with peroxynitrite, and both fully prevented the increase in DCF fluorescence induced by the combination of the NO^•^ donor and PTIO without any filter effect on the fluorescence of DCF (Figure 3B,C right panel). Therefore, H_2_DCF efficiently competed with other intracellular targets for ${\mathrm{NO}}_{2}^{•}$. In the experiments conducted with 15 µM sperNO, the rate at which the fluorescence increased slowed down toward the end of the run. The reason for this behavior was unclear, but it could involve exhaustion of H_2_DCF or further oxidation of the product DCF by ${\mathrm{NO}}_{2}^{•}$ [43], although the rate constant for the reaction between DCF and ${\mathrm{NO}}_{2}^{•}$ is two orders of magnitude lower than the reaction between DCF and the reduced PTIO.

### 3.5. Removal of the LIP by Chelation Increases Peroxynitrite-Dependent Intracellular Oxidation

We continued by repeating the critical experiments from our previous study, which had demonstrated augmented peroxynitrite-dependent intracellular oxidation of H_2_DCF upon chelation of the LIP in RAW 264.7 cells. We assessed the effect of the LIP by comparing the intracellular oxidation of H_2_DCF by PQ/NO^•^ with and without SIH, a cell membrane-permeable iron chelator that accesses the cytosolic space and binds to the LIP within a few minutes [2]. SIH is known for rapidly and strongly (Logβ_2_ = 26.9) binding ferrous iron in a 2:1 stoichiometry, yielding the (Fe(SIH)_2_) complex. This iron complex (Fe^3+^/Fe^2+^ redox pair at pH 7.4 is 0.13 V vs. NHE) does not undergo redox cycle to produce oxidants [58] and does not appear to react with peroxynitrite directly [23]. Direct oxidation of targets by peroxynitrite seems to take place by the inner-sphere mechanism [31,32,59], so metal chelators that prevent peroxynitrite binding are also likely to prevent the direct oxidation of transition metals by peroxynitrite.

To reproduce our previous experiments, we transferred a suspension of H_2_DCF-DA- loaded RAW 264.7 cells to 96-well plates and exposed it to peroxynitrite fluxes generated by PQ/NO^•^ as described above. As shown in Figure 4A, we recorded the fluorescence of DCF in real-time. We used the linear regression of the fluorescence data obtained during the first 10 min of each run to express these data as the rate of the increase in fluorescence, or the rate at which DCF was formed (Figure 4B). Importantly, the PQ/NO^•^-induced oxidation of H_2_DCF was negligible in the absence of NO^•^ and was fully inhibited in experiments performed in the presence of Ebselen (Figure 4A,B). Together, these observations indicated that peroxynitrite mediated the formation of DCF in the presence of PQ/NO^•^.

Consistent with our previous results [23], the rate at which DCF was formed increased significantly in the presence of SIH (Figure 4A,B), leading to the conclusion that LIP chelation increases the availability of peroxynitrite and its derived radical oxidants (Scheme 1). Importantly, in the absence of cells, SIH has no effect on peroxynitrite-dependent oxidation of H_2_DCF [23]. Together, these results lead to the conclusion that cellular SIH effect on peroxynitrite-dependent H_2_DCF oxidation is due to chelation of cellular LIP. In our previous study, we found that the cell membrane-permeable classic iron chelator 2,2′-bipyridine also elicits the chelator effect [23], indicating that this effect is not specific to SIH, but rather a common property of membrane-permeable LIP chelators. Finally, the oxidation of H_2_DCF and the enhanced effect of the chelators were also observed with endogenous NO^•^ produced by LPS-activated RAW 264.7 cells [23].

We estimated that the intracellular accumulation of DCF increased from 30 μM in the absence of SIH to 50 μM in the presence of SIH in the 60-min runs (Supplementary Material). This represented roughly 10% of the total intracellular concentration of H_2_DCF. It is also safe to assume that the total intracellular peroxynitrite produced during this time interval was at least 300 μM given that most peroxynitrite reacts with metalloproteins, thiol peroxidases, and other targets [46]. Given that LIP concentration in RAW 264.7 cells was 2 μM, LIP is the limiting species in its reactions with peroxynitrite.

Remarkably, Ebselen also fully prevented the formation of DCF in the presence of SIH, indicating that the oxidation of H_2_DCF in the presence of the chelator also depended on peroxynitrite. The fact that Ebselen (Figure 3C) and SIH (Figure 3A,C left panel) failed to affect the ${\mathrm{NO}}_{2}^{•}$-dependent oxidation of H_2_DCF excluded the possibility that NO^•^ autoxidation was an important source of ${\mathrm{NO}}_{2}^{•}$ for the intracellular oxidation of H_2_DCF under our experimental conditions. Indeed, SIH and Ebselen can neither prevent the formation of ${\mathrm{NO}}_{2}^{•}$ from NO^•^ autoxidation nor efficiently compete with H_2_DCF for ${\mathrm{NO}}_{2}^{•}$ generated from NO^•^ and PTIO (PTIO is cell membrane-impermeable and thus generates ${\mathrm{NO}}_{2}^{•}$ extracellularly, a compartment where both SIH and Ebselen may react with ${\mathrm{NO}}_{2}^{•}$, explaining why the oxidation of intracellular H_2_DCF is partially inhibited in the presence of SIH or Ebselen.) (Figure 3) as shown here. Together with the observations that SIH increased and Ebselen prevented the PQ/NO^•^- induced oxidation of H_2_DCF, these results showed that the intracellular oxidants of H_2_DCF generated by treatment with PQ/NO^•^ primarily derived from peroxynitrite ([23]) under the study conditions. Finally, the fact that SIH failed to increase the formation of DCF in cells exposed to NO^•^ and PTIO (this failure does not mean that the LIP does not react with ${\mathrm{NO}}_{2}^{•}$; it probably does react, but other ${\mathrm{NO}}_{2}^{•}$ endogenous targets such as GSH (5–10mM) and exogenous targets such as H_2_DCF (400 µM) outcompete the LIP (2 µM) for ${\mathrm{NO}}_{2}^{•}$) provided further evidence to refute the possibility that free SIH or the [Fe(SIH)_2_] complex somehow produces oxidants that would stimulate the formation of DCF inside the cells.

**Figure 5:**
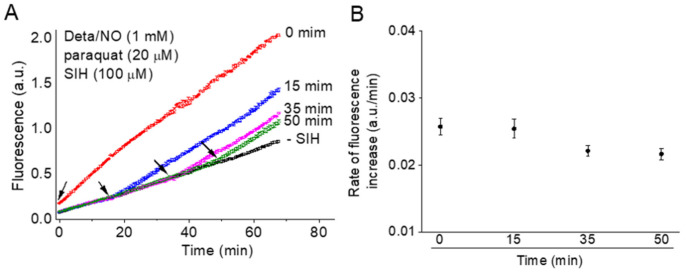
The rate of intracellular oxidation of H_2_DCF as a function of the time when SIH was added. (**A**). Fluorescence traces due to the formation of DCF. The experiments were performed as described in Figure 4; 2,2′-(hydroxy-nitroso-hydrazono)bis-ethanimine (Deta/NO) was used as NO^•^ donor in the presence of paraquat. SIH was added before or at selected time points during the run, as designated in the figure. Deta/NO has a long half-life under the experimental conditions, producing a constant flux of NO^•^ in the time scale of the experiments. The data represent the mean ± S.D. of four experiments; (**B**). The rate at which the fluorescence increased after SIH was introduced. The rate was determined by linear regression of the fluorescence traces in the final 5 min of the collected data. The data represent the mean of four independent experiments ± S.D. The conditions and fluorescence acquisition and parameter settings were the same as those described in Figure 3.

Other possibilities could explain the enhanced oxidation of H_2_DCF in the presence of SIH. However, extensive control experiments conducted in our previous study [23] showed that: (i) SIH and the [Fe(SIH)_2_] complex, which mimics the SIH/ferrous LIP, does not produce $O\frac{•}{2}$ or other oxidants; (ii) neither the free SIH nor the [Fe(SIH)_2_] complex suppresses or interferes in the fluorescence properties of DCF; (iii) SIH does not interfere with the concentration of NO^•^ in cells; (iv) neither SIH nor the [Fe(SIH)_2_] complex appears to react with peroxynitrite directly; (v) significant H_2_O_2_-dependent oxidation of H_2_DCF did not happen under the experimental conditions of the study. Indeed, almost no DCF was formed in the absence of NO^•^ (indistinguishable from control experiments) and in the presence of PQ, a condition in which the availability of $O\frac{•}{2}$ and H_2_O_2_ would be higher (Figure 4) [23]. This observation indicated that the chemistry of H_2_O_2_ with the LIP or iron peroxidases is not relevant for the intracellular oxidation of H_2_DCF under the study conditions. The chelator SIH also attenuates the H_2_O_2_-dependent oxidation of H_2_DCF [18] and increases the PQ/NO^•^-dependent oxidation of H_2_DCF in RAW 264.7 cells (Figure 4) [23], indicating that the chemical mechanisms for the oxidation of H_2_DCF by H_2_O_2_ and PQ/NO^•^ in RAW 264.7 cells are fundamentally different; (vi) there is no reason to believe that SIH changes the concentrations of antioxidants in cells that prevent the formation of peroxynitrite (e.g., SODs) or that compete with H_2_DCF for peroxynitrite-derived radicals (e.g., GSH). The chelator enhancement effect is observed within seconds to few minutes after the chelator is introduced (please, see Figure 5A below), a time scale that is insufficient to affect the concentrations of the antioxidants. This time scale, however, is compatible with the kinetics of SIH cell membrane permeability and binding to the LIP (Figure 1A) [2,17]; (vii) H_2_DCF is oxidized in the presence of NO^•^ alone (in the absence of PQ), but this is possibly related to the formation of peroxynitrite by reaction of NO^•^ with endogenous $O\frac{•}{2}$ since H_2_DCF was also inhibited by Ebselen (not shown).

### 3.6. Catalytic Properties of the Reaction between the LIP and Peroxynitrite

One important missing feature of the reaction between the LIP and peroxynitrite is whether it is stoichiometric or catalytic. We estimated that the concentration of the LIP in RAW 264.7 cells was 2 μM, and that at least 300 μM of peroxynitrite was formed in cells exposed to PQ/NO^•^ in the period of 60 min, so we concluded that the LIP was the limiting reactant of the reaction between the LIP and peroxynitrite under the experimental conditions of the study. We rationalized that if the reaction between the LIP and peroxynitrite were stoichiometric, the LIP would be gradually consumed by the reaction with peroxynitrite and thus its concentration would be reduced with delayed addition of SIH. In fact, we rationalized that the rate at which DCF is formed in the absence of the chelator would actually increase, approaching the rate at which DCF was generated in the presence of the chelator (labeled as 0 min in the Figure below). However, accumulated data [23,60] and results of the current study (Figure 4) showed that the rate at which DCF was formed in the absence of SIH was constant even at the highest concentration of NO^•^ donor used in the experiments. Assuming that the formation of peroxynitrite by PQ/NO^•^ was constant, as suggested by the CBA experiment (Figure 2), the concentration of the LIP form reactive toward peroxynitrite did not appreciably decrease over time, indicating that the LIP may catalytically remove peroxynitrite.

We tested the catalytic versus stoichiometric behavior of the reaction between the LIP and peroxynitrite by exposing the cells to peroxynitrite fluxes, as usual, but we introduced the chelator SIH at different time points during the experimental run (Figure 5A). Again, if the reaction between the LIP and peroxynitrite were stoichiometric, the SIH effect would gradually decrease due to consumption o LIP by peroxynitrite. However, as shown in Figure 5B, the rate at which the fluorescence increased after SIH was added remained unchanged regardless of the delay in the addition of SIH. Together, these observations suggested that the LIP catalytically reacts with peroxynitrite.

### 3.7. The Rate Constant of the Reaction between the LIP and Peroxynitrite

We estimated the rate constant for the hypothetical reaction between the LIP and peroxynitrite by using a rather simple reaction model (Equations (2)–(9)) for the rate of the peroxynitrite-dependent DCF formation, the parameter that we measure when we use the H_2_DCF assay in cells.

(2) $$O\frac{•}{2}+\mathrm{SOD}\;\overset{{\;k}_{2\;}}{\to }O_{2}$$

(3) $${\mathrm{NO}}^{•}+O\frac{•}{2}\overset{\;k_{3\;}}{\to }\;{\mathrm{ONOO}}^{-}$$

(4) $${\mathrm{ONOO}}^{-}+{\mathrm{CO}}_{2}\overset{{\;k}_{\;4}\;}{\to }\;0.35\;\left({\mathrm{NO}}_{2}^{•}+\mathrm{CO}\frac{•}{3}\right)+0.65\;\left({\mathrm{NO}}_{3}^{-}+{\mathrm{CO}}_{2}\right)$$

(5) $${\mathrm{ONOO}}^{-}+\mathrm{TP}\;\overset{{\;k}_{5}\;}{\to }\;\mathrm{OxTP}\;+{\mathrm{NO}}_{2}^{-}$$

(6) $${\mathrm{ONOO}}^{-}+\mathrm{LIP}\;\overset{{\;k}_{6}}{\to }\;{\;\mathrm{LIP}-\mathrm{Fe}}^{4+}O+{\mathrm{NO}}_{2}^{-}$$

(7) $$R^{•}+\mathrm{CC}\;\overset{{\;k}_{7\;}}{\to }{\;R}^{-}\;+\;{\mathrm{CC}}^{•}$$

(8) $$R^{•}\;+\;H_{2}\mathrm{DCF}\;\overset{{\;k}_{8\;}}{\to }\;R^{-}\;+\;{\mathrm{DCFH}}^{•}$$

(9) $${\mathrm{DCFH}}^{•}\;+\;O_{2}\;\overset{{\;k}_{9}\;}{\to }\;O\frac{•}{2}\;+\;\mathrm{DCF}$$

The consumption of $O\frac{•}{2}$ by SOD is simplified as the $O\frac{•}{2}$ oxidase reaction (Equation (2)), and the formation of peroxynitrite is represented by the co-reaction between ${\mathrm{NO}}^{•}$ and $O\frac{•}{2}$ Equation (3). Due to the inherent reactivity of peroxynitrite and its derived radicals, they can react with various cellular targets. All the direct reactions of peroxynitrite other than its reactions with CO_2_ Equation (4), $k_{4}$ = 3 × 10^4^ M^−1^s^−1^ [61,62], and the LIP (Equation (6)) and all the reactions of the peroxynitrite-derived radicals other than their reaction with H_2_DCF Equation (8) are represented generically in Equations (5) and (7), respectively). In Equation (5), TP stands for thiol and iron peroxidases/proteins, and oxTP represents the oxidized form of these species. The constant $k_{5}$ represents an average weighted rate constant that depends on the concentration and reactivity of the TP members, which vary among cells (we assume these values range from 1 × 10^5^ to 1 × 10^7^ M^−1^s^−1^) [63,64]. In Equation (7), CC and CC^•^ stand for a collection of cell constituents that can be oxidized by peroxynitrite-derived radicals (such as GSH) and their corresponding oxidized forms. The peroxynitrite-derived radicals are represented collectively as R^•^, and their corresponding reduced anions are represented as R^−^ (Equations (7) and (8)).

Assuming steady-state conditions for peroxynitrite and all the intermediate reactive species, our model demonstrated that the rate of the peroxynitrite-dependent indirect intracellular formation of DCF in the absence of the chelator is given by Equation (10) (see Supplementary Material for the demonstration of the equation).

(10) $$\frac{d\;[\mathrm{DCF}]}{\mathrm{dt}}=\frac{2/{3\;k}_{3}{\;k}_{4}{\;k}_{8}{[\mathrm{NO}}^{•}][O\frac{•}{2}][{\mathrm{CO}}_{2}]\;\left[H_{2}\mathrm{DCF}\right]}{{(k}_{4}{\;[\mathrm{CO}}_{2}]\;+\;k_{5}{\;[\mathrm{TP}]+k}_{6}{\;[\mathrm{LIP}])\;(k}_{7}\;\left[\mathrm{CC}\right]\;+\;k_{9}\left[H_{2}\mathrm{DCF}\right])}$$

The equation shows that the rate of the oxidation of H_2_DCF depends directly on the concentrations of ${\mathrm{NO}}^{•}\;\mathrm{and}\;O\frac{•}{2}$ and indirectly on the concentration of TP, CC, and the LIP, while it has a complex relationship with the concentrations of CO_2_ and H_2_DCF. In the presence of the chelator, the rate of the oxidation of H_2_DCF is

(11) $$\frac{d\;[\mathrm{DCF}]}{\mathrm{dt}}=\frac{2/{3\;k}_{3}{\;k}_{4}{\;k}_{8}{[\mathrm{NO}}^{•}][O\frac{•}{2}]\left[{\mathrm{CO}}_{2}\right]\;\left[H_{2}\mathrm{DCF}\right]}{{(k}_{4}{\;[\mathrm{CO}}_{2}]\;+\;k_{5}{\;[\mathrm{TP}])\;(k}_{7}\;\left[\mathrm{CC}\right]\;+\;k_{9}\;\left[H_{2}\mathrm{DCF}\right])}$$

Because the term *k*_6_[LIP] vanishes from Equation (10).

**Figure 6:**
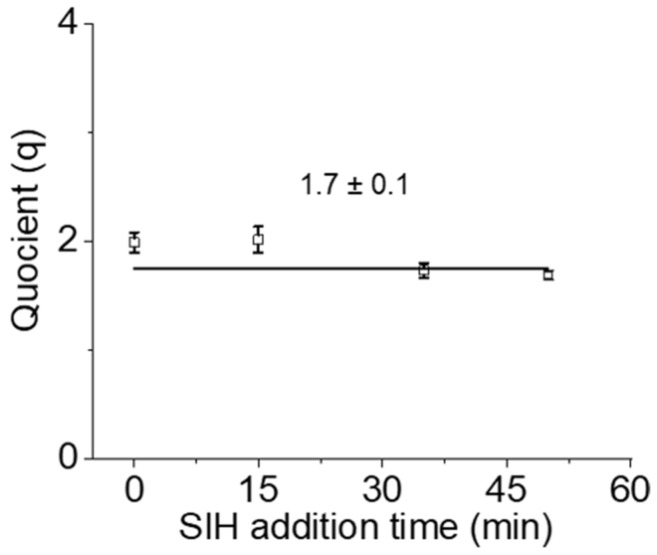
The quotient of the rates of fluorescence increased in the presence and absence of SIH. The quotient (q) was determined by dividing the rates of the increase in fluorescence in the presence of SIH by the rates of the increase in fluorescence in the absence of SIH; the data from Figure 5 were used.

Division of Equation (11) by Equation (10) and rearrangement gives an expression for *k*_6_ Equation (12), the rate constant for the hypothetical reaction between the LIP and peroxynitrite. This equation allows *k_6_* to be estimated if all the other parameters (q, *k*_4,_ *k*_5,_ and the concentrations of CO_2_, TP, and LIP) are known. We determined the quotient (q), which represents a dimensionless parameter for the enhanced effect of the chelation of the LIP on the rate of the peroxynitrite-dependent indirect formation of DCF, experimentally. Using the data from Figure 5, we calculated the mean value of q as 1.7 (Figure 6). Whereas we did not measure the concentration of CO_2_ experimentally, its concentration in the aqueous phase of cellular suspensions under similar conditions (i.e., bicarbonate free buffer and open vessels) and experimental time scales suggest concentrations of CO_2_ in the range of hundreds of micromolar [65]. The concentration of TP varies depending on the cell type and cellular compartments. Considering only the thiol peroxidases, which are by far the most abundant TP members [66,67] and according to Wang et al. [68] and some calculations, TP concentration in several mammalian cell lines like *Mus musculus* and Raw 264.7 cells is around 10 μM. We determined that the concentration of the LIP in RAW 264.7 cells was 2.0 μM (Figure 1).

(12) $$k_{6}=(q\;-\;1)\;\frac{{\;k}_{4}\;\left[{\mathrm{CO}}_{2}\right]{+k}_{5\;}\left[\mathrm{TP}\right]}{\left[\mathrm{LIP}\right]}$$

Thus, assuming the concentrations of CO_2_ [65] and TP as 100 and 10 µM, respectively, as well as the lower and higher end limits of *k*_5_ (10^5^–10^7^ M^−1^s^−1^), we estimated the rate constant for the hypothetical reaction between peroxynitrite and the ferrous LIP to be between 1.4 × 10^6^ M^−1^s^−1^ and 40 × 10^6^ M^−1^s^−1^ in RAW 264.7 cells.

We recognize that our approach has several limitations and uncertainties. Nevertheless, we think that our assumptions are justified (please see below). In addition, the estimated range for the rate constant of the reaction between the ferrous LIP and peroxynitrite is comparable to the rate constants determined for similar reactions involving a two-electron oxidation of the ferrous ions of deoxymyoglobin [31,32] or myeloperoxidase [30] by peroxynitrite, for which k is in the order of 10^6^ M^−1^s^−1^. The uncertainties of the model may include: (i) the fact that the model assumed that any other mechanism for the oxidation of H_2_DCF other than the mechanism mediated by peroxynitrite (through its derived radicals) are equal in the absence and presence of chelator. These may include the oxidation of H_2_DCF by reactions of H_2_O_2_ with iron proteins and with metallo-peroxidase. Under our experimental conditions, however, the formation of DCF fully depended on the availability of NO^•^ and could be prevented by the peroxynitrite scavenger Ebselen, so the H_2_O_2_-dependent formation of DCF was apparently not significant; (ii) similarly, our model assumed that the pathways for the consumption of $O\frac{•}{2}$ (cellular SODs) and NO^•^ (cellular consumption of NO^•^and autoxidation of NO^•^) are equal in the absence and presence of chelator; (iii) the concentrations of TP and CO_2_ we used for the calculations are based on the literature, but given the simplicity of the model and the uncertainties above, determining these concentrations would not significantly increase the accuracy of the result; (iv) the model does not consider that the chelator itself is oxidized by the peroxynitrite-derived radicals and competes with H_2_DCF for these oxidants, which would have the practical effect of artefactually attenuating the incremental effect of the chelator on the rate at which DCF is formed.

## 4. Discussion

The nature of the cellular ferrous LIP remains unclear, but it is usually assumed to consist of small-molecular-mass iron complexes. On the basis of the available thermodynamic constants and concentrations, among all the bioavailable small molecular cellular ligands, only GSH could potentially serve as a meaningful ligand for the ferrous LIP in cells [69,70]. The reaction of GSH with either Fe(III) or Fe(II) ions in solution within the pH range of 3–7 yields the Fe(II)-GS complex only (excess GSH reduces ferric to ferrous iron), and empirical evidence supports the formation of Fe(II)-GS complexes in the cytosol [69,70]. However, we have hypothesized that the LIP is protein-bound by rationalizing that proteins potentially offer multiple ligands to iron [17], essentially functioning as metal chelator and forming species with higher thermodynamic affinity constants than GSH. This hypothesis is also based on the observation that iron in mononuclear non-heme enzymes such as prolyl hydroxylases (PHDs) are chelatable [71]. Additionally, a recent and convincing set of studies have strongly suggested that the LIP is bound to polyr(C)-binding protein 1 and 2 (PCBP1 and PCBP2) and possibly to other members of the PCBP family [72,73]. These proteins were initially characterized as RNA-binding proteins, but human PCBP1 and PCBP2 have been suggested to bind and to deliver iron to ferritin (the iron storage protein) [74], to deliver iron to cytosolic non-heme enzymes with mono- and di-nuclear iron centers such as prolyl hydroxylases and deoxy-hypusine hydroxylase (DOHH) [75,76], and to bind to mammalian protein BolA2, which together with gluta-redoxin 3 functions as (2Fe-2S) chaperones [72,73]. Conceptually, PCBP1 and PCBP2 have been suggested to be the LIP cellular ligands and major iron chaperones playing an integral roles in safe intracellular iron trafficking. The PCBP proteins are comprised of three discrete domains (called KH domains) [77]. Mutational analysis of the KH3 domain revealed that conserved cysteine and glutamate residues are necessary for PCBP1 to coordinate with iron [73]. Importantly, studies have shown that the purified recombinant PCBP1 KH3 binds Fe(II) with a dissociation constant of 4.2 and 0.8 μM in the absence and in the presence of 5 mM GSH, respectively [73], suggesting that GSH completes the coordination sphere of iron in PCBP proteins, reconciling the importance of GSH for the binding of the LIP in cells.

In this study, we focused on the fundamental properties of the reaction between peroxynitrite and the LIP in cells. Overall, the results presented herein indicate that the LIP directly, rapidly, and catalytically reacts with peroxynitrite in RAW 264.7 cells. Hypothetically, the ferrous LIP directly reduces peroxynitrite to nitrite (while it is oxidized to LIP-Fe=O^4+^), which presumably lowers the direct and indirect oxidation of cellular targets by peroxynitrite and its derived radicals. The reduction of LIP-Fe^4+^O back to the ferrous LIP would make the reduction of peroxynitrite by the LIP a catalytic process, which seems to be the case. The hypothetical LIP-Fe^4+^O reducing agent, the actual reactions, and the kinetic properties remain obscure, but given the possible PCBP1-GSH nature of the LIP, GSH seems to be a plausible candidate for the reduction of the LIP-Fe^4+^O species. Previous studies have demonstrated that the inhibition of the biosynthesis of GSH increases the oxidation of H_2_DCF [21] relative to the control in RAW 264.7 cells exposed to peroxynitrite, which is consistent with our hypothesis. However, these effects cannot be easily dissociated from direct scavenging of the peroxynitrite-derived oxidants by GSH. The depletion of GSH may also interfere in the PCBP-Fe(II) binding equilibrium and possibly with the reactivity of the LIP. Again, assuming the PCPB nature of LIP, it is also possible that protein amino acid residues act as the reducing agent for LIP-Fe^4+^O species. It is not unlikely that the Ferrous LIP and peroxynitrite reaction actually generates ferric LIP and ${\mathrm{NO}}_{2}^{•}$. Still, LIP could efficiently scavenge peroxynitrite anion while driving its oxidizing power into a single ferryl (LIP-Fe^4+^O) or radical (${\mathrm{NO}}_{2}^{•}$) pathway, which could then be more effectively neutralized by cellular reductants such as GSH and maybe ASC [78]. Nonetheless, the catalytic characteristics combined with the rate constant estimated for the reaction between the LIP and peroxynitrite (*k*_6_ = 1.4–40 × 10^6^ M^−1^s^−1^) suggest that the LIP may represent a relevant peroxynitrite reductase system in RAW 264.7 cells, efficiently competing with the reaction between peroxynitrite and CO_2_, which generates reactive radical oxidants. Given the ubiquity of LIP and that iron is a kinetic preferential target of peroxynitrite, it is tempting to conclude that the peroxynitrite and the LIP reaction is important in other cell types, especially in condition where peroxynitrite formation is likely such as infection and inflammation. However, this remains to be tested considering that the relevance of LIP at detoxifying peroxynitrite in cells depends on the concentration of the LIP itself and presumably on the activity of other peroxynitrite reductases systems that compete with the LIP for the oxidant, such as peroxiredoxins and glutathione peroxidases; the higher their concentration/activity the lower the LIP relevance (Equation (12)). Thus, the peroxynitrite reductase activity of LIP is probably cell type dependent.

The finding that the LIP attenuates the peroxynitrite-dependent oxidation apparently contradicts the documented pro-oxidant properties of the LIP. We extensively discussed this issue in our previous work [23]. It is important to point out again that the redox toxicity of the LIP is often rationalized in terms of its reaction with H_2_O_2_, and that the pro-oxidant outcome of such interaction is not a shared feature with other peroxides. Furthermore, the toxic iron that accumulates in chronic conditions such as Hemochromatosis, Thalassemia Major, and brain disorders or trauma, ferroptosis [79,80] and in acute iron loading does not necessarily represent the LIP and may have different chemical properties, including different reactivity toward H_2_O_2_ and peroxynitrite. Kakhlon et al. [81] showed that iron associated with the LIP in K562 cells only demonstrably increased the hydrogen peroxide-dependent oxidation of proteins when the LIP levels were manipulatively raised to at least 2.6-fold above its basal conditions, suggesting that LIP is not an oxidant while bound to its cellular ligand, but that the species binding to the LIP had limiting buffer capacity. The possible PCBP1 and other iron proteins nature of the LIP cannot be ignored, and it is not yet known how the expression of these proteins is affected under different conditions of iron overload. Thus, the relationship between the LIP and the toxic iron source that develops and accumulates under situations in which iron is dysregulated is not straightforward. Production of the oxyferryl oxidant species LIP-Fe^4+^O (Scheme 1, Equation (6)) by the hypothetical reaction between peroxynitrite and the LIP may also appear conflicting with our results. However, LIP-Fe^4+^ O is probably less reactive than peroxynitrite and its derived radical species. In addition, given the possible PCBP-GSH nature of the LIP, the LIP-Fe^4+^ O oxidative power may be neutralized by GSH.

One last point is worth discussing. We [17] and others [18] have shown that the LIP also reacts with NO^•^. The competitive nature of the reaction of the LIP with NO^•^ and with peroxynitrite is not surprising given that this is a shared property of iron compounds and iron proteins. We reason that the reaction of the LIP with NO^•^ or peroxynitrite depend on the conditions, notably the availability of $O\frac{•}{2}$. Possibly, the formation of DNIC predominates under normal conditions, while the reaction between the LIP and peroxynitrite gains importance with increasing levels of $O\frac{•}{2}$ given that the latter gradually outcompetes the LIP for NO^•^. In addition, the reaction of the LIP with NO^•^ producing DNIC is rapid, but it is a complex multi-step process that requires at least two reversible NO^•^ binding steps and the reduction of Fe(II) to Fe(I) [22,82], which may limit the formation of DNIC. Interestingly, the formation of DNIC prevents the LIP- and H_2_O_2_-dependent oxidation of H_2_DCF in RAW 264.7 cells [18]. Thus, with regard to the chemistry related to NO^•^ and peroxynitrite, the LIP appears to exhibit complementary antioxidant activities under normal and oxidative conditions although this occurs through fundamentally different chemical mechanisms.

## 5. Conclusions

In summary, the LIP rapidly and possibly catalytically reacts with peroxynitrite. This reaction potentially attenuates the modifications made by peroxynitrite at biological targets, as we had shown in the case of protein carbonylation [23]. These findings broaden the conventional view of the reactivity of the LIP and have potential physiologically relevant implications in redox biology, particularly in infection and inflammation conditions when peroxynitrite is likely to be formed.

## Data Availability

Not applicable.

## Supplementary Materials

The following are available online at https://www.mdpi.com/article/10.3390/biom11091331/s1, Quantification of total intracellular H_2_DCF (reduced) loaded into RAW264.7 cells upon treatment (Figure S1) and accumulated DCF after cell treatment with NO^•^ donor and paraquat. generation of fluxes of ${\mathrm{NO}}_{2}^{•}$ (Figure S2), model for the oxidation of H_2_DCF by peroxynitrite-derived radical oxidants (Equations (S1)–(S25)).
