# Supplementary material for: The Labile Iron Pool Reacts Rapidly and Catalytically with Peroxynitrite
Source: Biomolecules. 2021 Sep 9;11(9):1331. doi: 10.3390/biom11091331 (PMC8466499; doi:10.3390/biom11091331)
Supplement: Supplementary file 1 [file biomolecules-11-01331-s001.zip › biomolecules-1284106-supplementary.pdf]

Supplementary Material

Quantification of intracellular H_2DCF and accumulated DCF

Intracellular H_2DCF.

A suspension of RAW 264.7 cells in PBS/DTPA (60 × 10^6 cell/mL) was treated with 30 μM H_2DCF-DA at constant stirring and 37 ^0C for 30 min, double washed by two cycles of centrifugation and resuspension in ice-cold PBS/DTPA, and kept on ice. This step minimizes the presence of extracellular H_2DCF species. Then, the cells were lysed by three 20-second cycles of point sonication and centrifuged at 20,000 g, to sediment cell debris. The soluble fraction of the cell extract was saved for quantification of H_2DCF and kept on ice bath (total of 3 mL). Then, 200 μL of this cell extract was diluted in PBS/DTPA to a final volume of 1 mL. The absorption spectrum of this solution is shown in figure S1A (labelled as cell extract). It revealed only a negligible amount of DCF after this procedure. The reduced H_2DCF in this solution was fully oxidized by addition of horseradish peroxidase (0.2 μM) and three successive additions of H_2O_2 (2 μM), which was accompanied by the expected increase in absorption at 502 nm (HRP + H_2O_2). No changes were observed after the first addition of hydrogen peroxide.

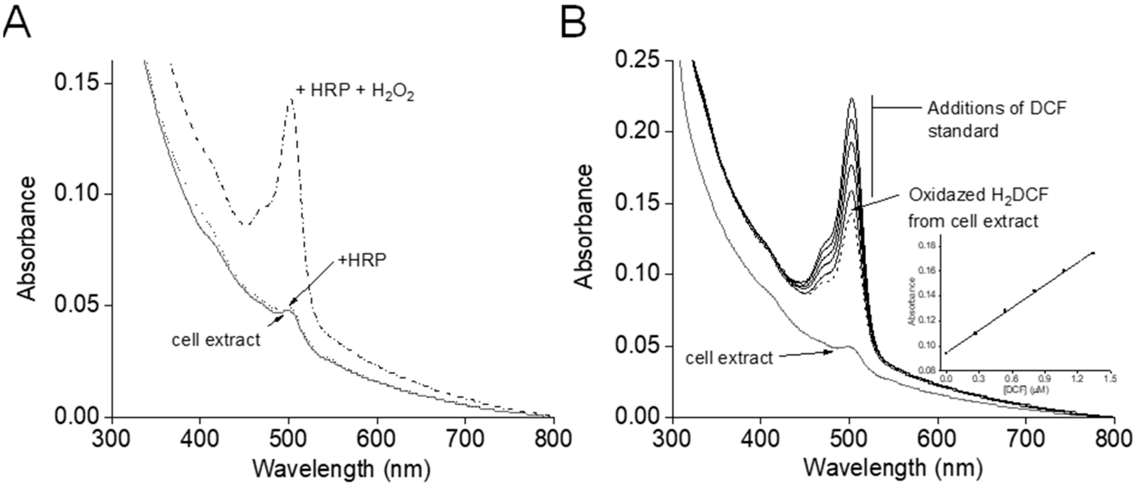

Figure S1. Quantification of total reduced intracellular H_2DCF. A) Spectra of the cell extract before and after the oxidation of H_2DCF. B) Addition of the internal standard addition. A standard solution of DCF (0.27 μM) was sequentially added to the resulting solution as shown in (B), inset; linear internal standard.

The total concentration of DCF in the solution was determined by the Beer-Lambert law using the difference between the absorption at 502 nm in the final and initial spectra (Figure S1-A) and the internal standard addition method (Figure S1-B and inset). The two methods returned essentially the same concentration for the totally reduced H_2DCF in solution (1.6 μM). The intracellular concentration of totally reduced H_2DCF was calculated as being 400 μM by unit conversion, by using the following equation: ([H_2DCF] × total volume of the assay/total volume of cells corresponding to 200 μL of cell extract). The diameter of the RAW 264.7 cells was taken as being 7 μm as reported elsewhere.

Accumulated intracellular concentration of DCF after treatment with PQ/$NO^•$.

The intracellular concentration of DCF accumulated in the experiments of Figure 2 (main text) was also estimated after the typical exposure of RAW 264.7 cells to PQ/$NO^•$ for 60 min. The cellular PQ/$NO^•$ experiments were performed as usual, in 96-well plates along with control (H_2DCF-unloaded) RAW 264.7 cells in the presence of increasing standard concentrations of DCF from the beginning. After exposure for 60 min, the final fluorescence signal of the DCF standards was used to build an analytical curve for DCF in the presence of the cells. This curve was used to determine the concentration of DCF accumulated in solution in the samples of cells exposed to PQ/$NO^•$ with or without SIH for 60 min. The intracellular concentration of DCF in these cells was determined by unit conversion, as described above, being 30 and 50 μM in the absence and presence of SIH, respectively.

Generation of fluxes of $NO_2^{\bullet}$

To test whether H_2DCF reacted with peroxynitrite-derived radicals under our experimental conditions, it was necessary to design a strategy to specifically deliver these species to the cellular suspension model. $NO_2^{\bullet}$ was selected as the prototype species, and fluxes of this compound were generated by mixing a $NO^{\bullet}$ donor and 2-phenyl-4,4,5,5-tetramethylimidazoline-1-oxyl 3-oxide (PTIO), an imidazolineoxyl N-oxide that oxidizes $NO^{\bullet}$ to $NO_2^{\bullet}$ [1, 2] (eq. 1). To ensure that the RAW 264.7 cells loaded with H_2DCF were exposed to a continuous flux of $NO_2^{\bullet}$, first the oxidation of H_2DCF and the nitrosylation of 4,5-diaminofluorescein (DAF2)[3] were followed in the presence of sperNO and increasing concentrations of PTIO. This step was important to determine the concentration of PTIO that was necessary for oxidizing almost all of the $NO^{\bullet}$ to $NO_2^{\bullet}$ under our experimental conditions.

$$PTIO^{\bullet} + NO^{\bullet} \xrightarrow{k=1\times10^{4}M^{-1}s^{-1}} PTI^{\bullet} + NO_2^{\bullet} \qquad (1)$$

According to us[4] and Espey at al.[5], the nitrosylation of DAF2 (eqs. 2-3) takes place primarily by a two-step radical process (oxidative nitrosylation) involving the oxidation of the indicator to a putative DAF2 radical (eq. 2) that, in turn, recombines with $NO^{\bullet}$ and condenses to produce water and the fluorescent triazol product DAFT (eq. 3). Low concentrations of PTIO accelerate the oxidation of $NO^{\bullet}$ to $NO_2^{\bullet}$ relative to $NO^{\bullet}$ autoxidation (eq. 1) while still leaving enough free $NO^{\bullet}$ for the rapid recombination reaction that ultimately leads to DAFT (eq. 3), consequently increasing the rate of the nitrosylation of DAF2[5]. Higher concentrations of PTIO, however, repress the rate at which DAF2 is nitrosylated due to increased $NO^{\bullet}$ scavenging and prevention of reaction 3. The results of the experiments performed with RAW 264.7 cells loaded with DAF2 were consistent with this interpretation given that 200 μM and higher concentrations of PTIO brought the rate at which DAFT was formed to control levels (Figure S2A)

$$DAF2 + NO_2^{\bullet} \rightarrow DAF2^{\bullet} + NO_2^{-} \qquad (2)$$

$$DAF2^{\bullet} + NO^{\bullet} \rightarrow DAFT + H_2O \qquad (3)$$

The rate at which H2DCF was oxidized, on the other hand, increased and reached a plateau as expected from the oxidation of $NO^{\bullet}$ to $NO_2^{\bullet}$ with increasing concentration of PTIO (Figure S2B). Therefore, at 200 μM PTIO, the nitrosylation of DAF2 decreased to a minimum and the oxidation of H2DCF increased to a maximum, thus suggesting that 200 μM PTIO maximized $NO_2^{\bullet}$ production from sperNO and significantly depleted $NO^{\bullet}$. In addition, on the basis the rate at which DCF was formed, different $NO_2^{\bullet}$ fluxes were generated by varying the concentration of the $NO^{\bullet}$ donor used at the same concentration of PTIO (Figure S2B).

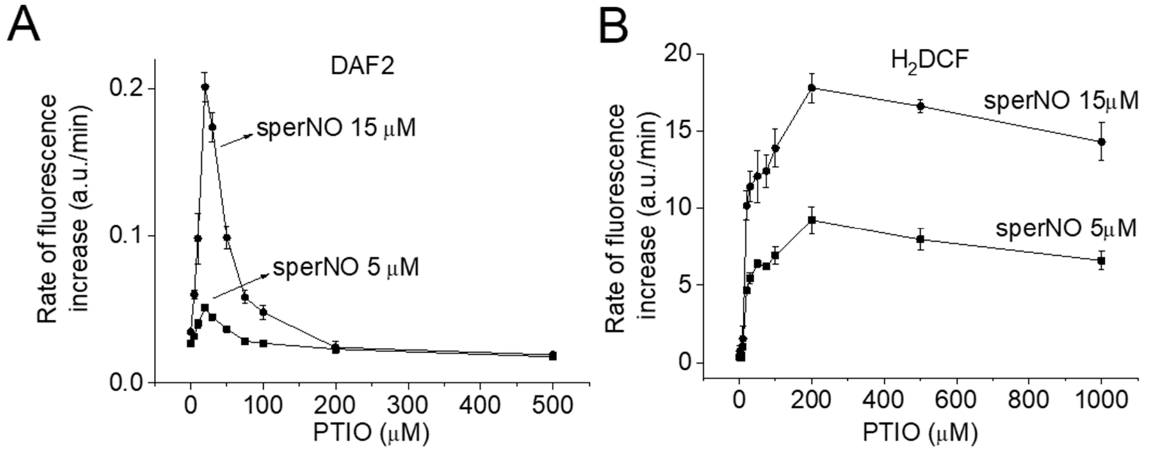

Figure S2. The effect of increasing concentration of PTIO on the rate of the $NO^{\bullet}$-dependent nitrosylation of DAF2 and oxidation of H2DCF in cells. Rate of intracellular nitrosylation of DAF2 (A) and rate of intracellular oxidation of H2DCF (B) by the combination of sperNO and PTIO. A suspension of DAF2- or H2DCF-loaded RAW 264.7 cells was transferred to 96-well plates (1.2×10^7 cells/mL) and treated with sperNO (5 or 15 μM) and increasing concentrations of PTIO as indicated in the plots. The fluorescence was measured immediately after sperNO was introduced and registered every minute for at least one hour. The rate was determined by using the approach described in the legend of Figure 2. The data represent the mean of four independent experiments ± S.D. The experimental conditions and fluorescence acquisition parameter settings for H2DCF were described in the legend of Figure 2. For the experiments with DAF2, the excitation (λex) and emission (λem) wavelengths were 495 and 520 nm, respectively.

PTIO can react with $NO_2^{\bullet}$, producing NO_2^- [6] and a putative PTIO cation radical that could potentially oxidize H2DCF in the experiments presented in Figure S2, but that possibility was ruled out. In fact, the rate at which DCF was formed decreased slightly at high concentrations of PTIO, which could be tentatively attributed to PTIO-mediated $NO_2^{\bullet}$ scavenging, excluding the possibility of PTIO and the PTIO cation radical being the oxidant of intracellular H2DCF. Additionally, PTIO[7] and possibly the putative PTIO cation radical are cell membrane-impermeable. Thus, $NO_2^{\bullet}$ was most likely the species accounting for the intracellular oxidation

of H_2DCF in the experiments in Figure S2B. This notion was strengthened by performing experiments in the presence of rapid $NO_2^{\bullet}$ scavengers, hexacyanoferrate (II) (FCN) [8, 9] and ascorbate (ASC^-) [10]. Neither FCN nor ASC^- reacts rapidly or at all with peroxynitrite, and both fully prevented the increase in DCF fluorescence induced by the combination of the $NO^{\bullet}$ donor and PTIO without any filter effect on the fluorescence of DCF (Main text Figure 3C, right panel).

The model for the oxidation of H_2DCF by peroxynitrite-derived radical oxidants

A simplified reaction model for the peroxynitrite-dependent intracellular oxidation of H_2DCF in cells is presented below. The model considers the flux (eq. 4) and the SOD consumption of $O_2^{\bullet -}$, represented simply by the $O_2^{\bullet -}$ oxidase reaction (eq. 5). Formation of peroxynitrite is represented as the co-reaction of nitric oxide ($NO^{\bullet}$) and the paraquat-derived $O_2^{\bullet -}$ (eq 6). The acid-catalyzed decomposition of $ONOO^-$ was assumed to be negligible as compared to its CO_2-catalyzed decomposition under our experimental conditions. Also, the autoxidation of $NO^{\bullet}$ was rationally and experimentally ruled out as a relevant source of $NO_2^{\bullet}$. Thus, all the peroxynitrite-derived radicals $NO_2^{\bullet}$ and $CO_3^{\bullet -}$ were assumed to originate from the reaction between peroxynitrite and CO_2 (eq. 7). These radicals are represented collectively as $R^{\bullet}$ ($NO_2^{\bullet} + CO_3^{\bullet -}$) (eqs. 7, 10, and 11). The steady-state concentrations of these radicals are probably very low, so dismutation and recombination reactions are justifiably irrelevant in cells.

As mentioned in the text, peroxynitrite displays reactivity toward various intracellular targets. All of the direct reactions with peroxynitrite other than with CO_2 (eq. 7) and the LIP (eq. 9) and all the reactions of peroxynitrite-derived radicals other than with H_2DCF (eq. 11) are represented generically (eqs. 8 and 10, respectively). TP includes peroxiredoxin, glutathione peroxidases, and iron peroxidases and proteins, while oxTP stands for the oxidized forms of these enzymes. CC stands for cell constituents (CC) that are susceptible to radical oxidation. Average rate constants were attributed to these generic reactions.

The model is only valid for the initial phase of the reaction given that the possible reduction reactions of the oxidized forms of the LIP and TP were omitted for simplicity. Nonetheless, the peroxynitrite-dependent intracellular oxidation of H_2DCF was constant throughout the experiments (Figure 4, from the main text), suggesting that the concentrations of the forms of TP and the LIP that are reactive toward peroxynitrite were in steady-state conditions, which allowed steady-state approximations to be use.

$$\xrightarrow{k_4} O_2^{\bullet-} \qquad (4)$$

$$O_2^{\bullet-} + SOD \xrightarrow{k_5} O_2 \qquad (5)$$

$$NO^{\bullet} + O_2^{\bullet-} \xrightarrow{k_6} ONOO^- \qquad (6)$$

$$ONOO^- + CO_2 \xrightarrow{k_7} 1/3\ R^{\bullet}(=NO_2^{\bullet} + CO_3^{\bullet-}) + 2/3\ (NO_3^- + CO_2) \qquad (7)$$

$$ONOO^- + TP \xrightarrow{k_8} OxTP + NO_2^- \qquad (8)$$

$$ONOO^- + LIP \xrightarrow{k_9} LIP\text{-}Fe{=}O^{4+} + NO_2^- \qquad (9)$$

$$R^{\bullet} + CC \xrightarrow{k_{10}} R^- + CC^{\bullet} \qquad (10)$$

$$R^{\bullet} + H_2DCF \xrightarrow{k_{11}} R^- + DCFH^{\bullet} \qquad (11)$$

$$DCFH^{\bullet} + O_2 \xrightarrow{k_{12}} O_2^{\bullet-} + DCF \qquad (12)$$

Dismutation of $DCFH^{\bullet}$ to produce H_2DCF and DCF was assumed to be negligible due to the low steady-state concentrations of $DCFH^{\bullet}$ and its rapid consumption through the reaction with O_2 (eq. 12, $k_{12} \approx 1 \times 10^9\ M^{-1}s^{-1}$). Under the study experimental conditions reaction 12 very likely produces negligible amounts of $O_2^{\bullet-}$ when compared to paraquat[1].

---

[1] Formation of $O_2^{\bullet-}$ through this mechanism is predicted on the basis of the one-electron oxidation of H_2DCF (eq. 11), in our case indirect peroxynitrite-dependent oxidation of H_2DCF, given that only negligible oxidation of H_2DCF was observed in the absence of $NO^{\bullet}$ (even in the presence of paraquat) or in the presence of Ebselen. A rough estimation of the fraction of paraquat-derived $O_2^{\bullet-}$ that contributes to the oxidation of H_2DCF can be made on the basis of the kinetic competition arguments. **Competition of SOD and $NO^{\bullet}$ for $O_2^{\bullet-}$:** Assuming 10 μM SOD and $k_5 = 2 \times 10^9\ M^{-1}s^{-1}$, the product k_5 [SOD] $= 2 \times 10^4\ s^{-1}$; assuming 200 nM for $NO^{\bullet}$ (15 μM

From the model, the rate at which DCF is formed intracellularly is given by eq. 13.

$$\frac{d\,[DCF]}{dt} = k_{12}[DCFH^{\bullet}][O_2] \qquad (13)$$

The rate law for $R^{\bullet}$ is given by eq. 14, where 2/3 is used as an adjustment that corrects for the yield of the combined formation of $NO_2^{\bullet}$ and $CO_3^{\bullet-}$ in eq. 7.

$$\frac{d[R^{\bullet}]}{dt} = 2/3\ k_7\,[ONOO^-][CO_2] - k_{10}[CC][R^{\bullet}] - k_{11}\,[H_2DCF][R^{\bullet}] \qquad (14)$$

The steady-state conditions for the concentration of $R^{\bullet}$ can be reasonably assumed in eq. 14 because it represents the combined reactive species that are unlikely to accumulate, which leads to eq. 15 when it is solved for the steady-state concentration of peroxynitrite.

$$[ONOO^-] = \frac{[R^{\bullet}]\,(k_{10}[CC] + k_{11}\,[H_2DCF])}{2/3k_7[CO_2]} \qquad (15)$$

The rate law for peroxynitrite can be expressed by eq. 16, which is derived from eqs. 6-9. Assuming steady-state conditions and solving for peroxynitrite, eq. 17 is obtained.

$$\frac{d\,[ONOO^-]}{dt} = k_6[NO^{\bullet}][O_2^{\bullet-}] - k_7\,[ONOO^-][CO_2] - k_8\,[ONOO^-][TP] - k_9\,[ONOO^-][LIP] \qquad (16)$$

$$[ONOO^-] = \frac{k_6[NO^{\bullet}][O_2^{\bullet-}]}{(k_7[CO_2] + k_8\,[TP] + k_9\,[LIP])} \qquad (17)$$

Now, equating eqs. 15 and 17 and solving for $R^{\bullet}$ gives an expression for the steady-state concentration of $R^{\bullet}$ (eq. 18).

---

sper/NO)[4], ($k_6 = 2 \times 10^{10}\ M^{-1}s^{-1}$), the product $k_6[NO] = 4 \times 10^3\ s^{-1}$; From these products, it can be calculated that about 20% of paraquat-derived $O_2^{\bullet-}$ reacts with $NO^{\bullet}$ to form peroxynitrite. **Competition between CO_2 and other cellular targets for peroxynitrite:** Assuming TP = 20 μM and $k_8 = 1 \times 10^6\ M^{-1}s^{-1}$ (k_8[TP] = 20 s^{-1}), 200 μM CO_2, and $k_7 = 6 \times 10^4\ M^{-1}s^{-1}$ (k_7 [CO_2] = 12 s^{-1}), about 37.5% of peroxynitrite reacts with CO_2. About 1/3 of the product of the reaction between peroxynitrite and CO_2 decays to radicals, 2/3 if we consider the combined formation of $NO_2^{\bullet} + CO_3^{\bullet-}$ (eq 7). **Competition between H_2DCF and cell constituents for peroxynitrite derived radicals**: Assuming H_2DCF 400 μM and $k_{11} = 2.6 \times 10^8\ M^{-1}s^{-1}$ for $CO_3^{\bullet-}$ (k_{11} [H_2DCF] = $1.04 \times 10^5\ s^{-1}$) and 5 mM GSH and $k = 5.3 \times 10^6\ M^{-1}s^{-1}$ (k [GSH] = $2.6 \times 10^4\ s^{-1}$), from these products, about 75% of $CO_3^{\bullet-}$ reacts with H_2DCF to yield $DCFH^{\bullet}$, the source of H_2DCF-derived $O_2^{\bullet-}$ according to eq. 12. From these successive fractions and the stoichiometry of the reactions, it is possible to calculate that only 6% of paraquat-derived $O_2^{\bullet-}$ ends up contributing to the oxidation of H_2DCF to $DCFH^{\bullet}$. That is, roughly, the H_2DCF-derived $O_2^{\bullet-}$ accounts for 6% of the paraquat-derived $O_2^{\bullet-}$. That figure drops to less than 1% if we assume $k_8 = 1 \times 10^7\ M^{-1}s^{-1}$.

$$[R^\bullet] = \frac{2/3 k_6 [NO^\bullet][O_2^{\bullet -}] k_7 [CO_2]}{(k_7 [CO_2] + k_8 [TP] + k_9 [LIP])\,(k_{10} [CC] + k_{11} [H_2DCF])} \quad (18)$$

The rate law for the DCFH$^\bullet$ radical is given by eq. 19.

$$\frac{d\,[DCFH^\bullet]}{dt} = k_{11}\,[R^\bullet][[H_2DCF] - k_{12}\,[DCFH^\bullet][O_2] \quad (19)$$

Steady-state conditions can be reasonably assumed for this radical, giving an expression for its concentration (eq. 20).

$$[DCFH^\bullet] = \frac{k_{11}\,[R^\bullet][H_2DCF]}{k_{12}\,[O_2]} \quad (20)$$

Substituting the solution of eq. 18 for the steady-state concentration of R$^\bullet$ in the equation above gives

$$[DCFH^\bullet] = \frac{2/3\, k_6 [NO^\bullet][O_2^{\bullet -}]\, k_7 [CO_2]\, k_{11} [H_2DCF]}{(k_7 [CO_2] + k_8 [TP] + k_9 [LIP])\,(k_{10} [CC] + k_{11} [H_2DCF])\,(k_{12} [O_2])} \quad (21)$$

Substituting this solution for the steady-state concentration of [DCFH$^\bullet$] in eq. 13 gives the rate at which DCF is formed in the absence of the chelator.

$$\frac{d\,[DCF]}{dt} = \frac{2/3\, k_6\,[NO^\bullet][O_2^{\bullet -}]\, k_7\,[CO_2]\, k_{11}\,[H_2DCF]}{(k_7 [CO_2] + k_8 [TP] + k_9 [LIP])\,(k_{10} [CC] + k_{11} [H_2DCF])} \quad (22)$$

According to eq. 22, the rate at which DCF is formed would increase with higher fluxes of NO$^\bullet$ and $O_2^{\bullet -}$ (thus flux of peroxynitrite). As shown in Figure 2 in the main text, this is indeed the case. It is also expected that the rate will depend on the rate of the reaction between peroxynitrite and CO_2, which generates the radicals, as well as on the rate of the reaction of peroxynitrite-derived radicals with H_2DCF (eq. 11), albeit not in such a trivial manner. Moreover, the equation is consistent with the expectation that the rate at which DCF is formed should inversely depend on the rate of the competitive reactions of peroxynitrite with cellular constituents (e.g., TP; eq 8 and the LIP; eq. 9) and also between peroxynitrite-derived radicals and cell constituents (eq. 10).

The rate at which DCF is formed in the presence of the chelator is

$$\frac{d\,[DCF]}{dt} = \frac{2/3\ k_6[NO^\bullet][O_2^{\bullet-}]k_7\,[CO_2]\,k_{11}\,[H_2DCF]}{(k_7\,[CO_2] + k_8[TP])\,(k_{10}\,[CC] + k_{11}\,[H_2DCF])} \tag{23}$$

given that the term k_9 [LIP] vanishes from eq. 22.

Now, dividing eq. 23 by eq. 22 yields the dimensionless parameter q, which represents the enhancement effect of the chelation of the LIP on the rate at which DCF is formed. This parameter can be experimentally determined by the ratio between the rates at which DCF is formed in the presence and absence of the chelator.

$$q = 1 + \frac{k_9\,[LIP]}{k_7\,[CO_2] + k_8\,[TP]} \tag{24}$$

Solving eq. 24 for k_9 yields an expression for the rate constant of the reaction between the LIP and peroxynitrite, eq. 25.

$$k_9 = (q - 1)\ \frac{k_7\,[CO_2] + k_8\,[TP]}{[LIP]} \tag{25}$$

The steady-state concentration of $O_2^{\bullet-}$ can also be assumed as being

$$[O_2^{\bullet-}] = \frac{k_4}{(k_5[SOD] + k_6[NO^\bullet])} \tag{26}$$

by using eqs. 4 – 6. Substituting this result for the concentration of $O_2^{\bullet-}$ in eq. 22 gives eqs. 27 and 28 for the rate at which DCF is formed in the absence and presence of the chelator, respectively

$$\frac{d\,[DCF]}{dt} =$$

$$\frac{k_4\,k_6\,[NO^\bullet]\ 2/3\ k_7\ [CO_2]\ k_{11}\,[H_2DCF]}{(k_5[SOD] + k_6\,[NO])\ \ (k_7\,[CO_2] + k_8\,[TP] + k_9\,[LIP])\,(k_{10}\,[CC] + k_{11}\,[H_2DCF])} \tag{27}$$

$$\frac{d\,[DCF]}{dt} =$$

$$\frac{k_4\,k_6\,[NO^\bullet]\ 2/3\ k_7\ [CO_2]\ k_{11}\,[H_2DCF]}{(k_5[SOD] + k_6\,[NO])\ \ (k_7\,[CO_2] + k_8\,[TP])\,(k_{10}\,[CC] + k_{11}\,[H_2DCF])} \tag{28}$$

Eq. 27 shows that the rate at which DCF is formed is inversely proportional to the concentration of SOD, as expected. Dividing eq. 28 by eq. 27 results in eq. 24.

References

1. Akaike, T.; Yoshida, M.; Miyamoto, Y.; Sato, K.; Kohno, M.; Sasamoto, K.; Miyazaki, K.; Ueda, S.; Maeda, H., Antagonistic action of imidazolineoxyl n-oxides against endothelium-derived relaxing factor .no through a radical reaction. *Biochemistry* **1993,** *32* (3), 827-832.

2. Akaike, T.; Maeda, H., Direct quantitation of nitric oxide released from cells using liposome-encapsulated PTIO. In *Biology of Nitric Oxide, Pt 5*, Moncada, S.; Stamler, J.; Gross, S.; Higgs, E. A., Eds. 1996; Vol. 10, pp 171-171.

3. Kojima, H.; Nakatsubo, N.; Kikuchi, K.; Kawahara, S.; Kirino, Y.; Nagoshi, H.; Hirata, Y.; Nagano, T., Detection and imaging of nitric oxide with novel fluorescent indicators: diaminofluoresceins. *Anal Chem* **1998,** *70* (13), 2446-53.

4. Damasceno, F. C.; Facci, R. R.; da Silva, T. M.; Toledo, J. C., Jr., Mechanisms and kinetic profiles of superoxide-stimulated nitrosative processes in cells using a diaminofluorescein probe. *Free Radical Biology and Medicine* **2014,** *77*, 270-280.

5. Espey, M. G.; Thomas, D. D.; Miranda, K. M.; Wink, D. A., Focusing of nitric oxide mediated nitrosation and oxidative nitrosylation as a consequence of reaction with superoxide. *Proceedings of the National Academy of Sciences of the United States of America* **2002,** *99* (17), 11127-11132.

6. Goldstein, S.; Russo, A.; Samuni, A., Reactions of PTIO and carboxy-PTIO with (NO)-N-center dot, (NO2)-N-center dot, and O-2(center dot). *Journal of Biological Chemistry* **2003,** *278* (51), 50949-50955.

7. Espey, M. G.; Miranda, K. M.; Thomas, D. D.; Wink, D. A., Distinction between nitrosating mechanisms within human cells and aqueous solution. *Journal of Biological Chemistry* **2001,** *276* (32), 30085-30091.

8. Forni, L. G.; Moraarellano, V. O.; Packer, J. E.; Willson, R. L., Nitrogen-dioxide and related free-radicals - electron-transfer reactions with organic-compounds in solutions containing nitrite or nitrate. *Journal of the Chemical Society-Perkin Transactions 2* **1986,** (1), 1-6.

9. Goldstein, S.; Czapski, G., Indirect oxidation of ferrocyanide by peroxynitrite - Evidence against the formation of hydroxyl radicals. *Nitric Oxide-Biology and Chemistry* **1997,** *1* (5), 417-422.

10. Kirsch, M.; de Groot, H., Ascorbate is a potent antioxidant against peroxynitrite-induced oxidation reactions - Evidence that ascorbate acts by re-reducing substrate radicals produced by peroxynitrite. *Journal of Biological Chemistry* **2000,** *275* (22), 16702-16708.
